# Assessment of genetic diversity of *Plasmodium falciparum* circumsporozoite protein in Sudan: the RTS,S leading malaria vaccine candidate

**DOI:** 10.1186/s12936-021-03971-0

**Published:** 2021-11-10

**Authors:** Nouh Saad Mohamed, Hanadi AbdElbagi, Ahad R. Elsadig, Abdalla Elssir Ahmed, Yassir Osman Mohammed, Lubna Taj Elssir, Mohammed-Ahmed B. Elnour, Yousif Ali, Mohamed S. Ali, Omnia Altahir, Mustafa Abubakr, Emmanuel Edwar Siddig, Ayman Ahmed, Rihab Ali Omer

**Affiliations:** 1 Department of Parasitology and Medical Entomology, Tropical Medicine Research Institute, National Centre for Research, Khartoum, Sudan; 2 Molecular Biology Unit, Sirius Training and Research Centre, Khartoum, Sudan; 3 El-Raqi Hospital, National University, Khartoum, Sudan; 4 Health Emergencies and Epidemics Control General Directorate, Sudan Federal Ministry of Health, Khartoum, Sudan; 5 Faculty of Medicine, EL-Neelain University, Khartoum, Sudan; 6 Department of the Integrated Vector Management (IVM), Federal Ministry of Health, Khartoum, Sudan; 7 Mycetoma Research Center, University of Khartoum, Khartoum, Sudan; 8 Pediatric Epidemiology, Clinic and Polyclinic for Child and Adolescent Medicine, Medical Faculty, University of Leipzig, Leipzig, Germany

**Keywords:** Malaria, *Plasmodium falciparum* circumsporozoite protein (PfCSP), Vaccine, RTS,S, Hapd, N-terminal, central repeats, and C-terminal regions

## Abstract

**Background:**

The currently used malaria vaccine, RTS,S, is designed based on the *Plasmodium falciparum* circumsporozoite protein (PfCSP). The *pfcsp* gene, besides having different polymorphic patterns, can vary between *P. falciparum* isolates due to geographical origin and host immune response. Such aspects are essential when considering the deployment of the RTS,S vaccine in a certain region. Therefore, this study assessed the genetic diversity of *P. falciparum* in Sudan based on the *pfcsp* gene by investigating the diversity at the N-terminal, central repeat, and the C-terminal regions.

**Methods:**

A cross-sectional molecular study was conducted; *P. falciparum* isolates were collected from different health centres in Khartoum State between January and December 2019. During the study period, a total of 261 febrile patients were recruited. Malaria diagnosis was made by expert microscopists using Giemsa-stained thick and thin blood films. DNA samples were examined by the semi-nested polymerase chain reaction (PCR). Single clonal infection of the confirmed *P. falciparum* cases, were used to amplify the *pfcsp* gene. The amplified amplicons of *pfcsp* have been sequenced using the Sanger dideoxy method. The obtained sequences of *pfcsp* nucleotide diversity parameters including the numbers of haplotypes (Hap), haplotypes diversity (Hapd), the average number of nucleotide differences between two sequences (p), and the numbers of segregating sites (S) were obtained. The haplotype networks were constructed using the online tcsBU software. Natural selection theory was also tested on *pfcsp* using Fuand Li’s D, Fuand Li’s F statistics, and Tajima’s D test using DnaSP.

**Results:**

In comparison with the different *pfcsp* reference strains, the Sudanese isolates showed high similarity with other African isolates. The results of the N-terminal region showed the presence of 2 different haplotypes with a Hapd of 0.425 ± 0.00727. The presence of the unique insertion of NNNGDNGREGKDEDKRDGNN was reported. The KLKQP motif was conserved in all the studied isolates. At the central repeat region, 11 haplotypes were seen with a Hapd of 0.779 ± 0.00097. The analysis of the genetic diversity in the C-terminal region showed the presence of 10 haplotypes with a Hapd of 0.457 ± 0.073. Several non-synonymous amino acids changes were also seen at the Th2R and the Th3R T-cell epitope regions including T317K, E317K, Q318E, K321N, I322K, T322K, R322K, K324Q, I327L, G352N, S354P, R355K, N356D, Q357E, and E361A.

**Conclusions:**

In this study, the results indicated a high conservation at the *pfcsp* gene. This may further contribute in understanding the genetic polymorphisms of *P. falciparum* prior to the deployment of the RTS,S vaccine in Sudan.

## Background

Malaria continues to be a major global health problem, particularly in Africa where *Plasmodium falciparum* is the most prevalent species. In 2019, the World Health Organization (WHO) estimated about 229 million cases and 409 thousand deaths worldwide, with 95% of malaria occurrence to be reported from Africa [1]. In Sudan, malaria is a serious public health problem and over 75% of the country population at risk of the disease [1]. The global 2016–2030 technical strategy for malaria aims to achieve 90% reduction in malaria incidences by 2030 worldwide [2]. The systematic fight against malaria consist of enhanced surveillance and monitoring, vector control, case-detection and treatment in cooperation with improved diagnosis and treatment capacity [3] yet there are several rapidly developing challenges for this approach mainly including the emerging resistance for drugs and insecticides, and the limited resources and diagnostic capacity in the endemic countries. However, effective vaccine will offer a powerful alternative that might make a breakthrough in this fight [2].

Malaria vaccine has been recommended as one of the pillars for the successful malaria eliminate [2, 4]. In the last two decades, a newly designed vaccine to encounter *P. falciparum* malaria infection was developed based on the *P. falciparum* circumsporozoite protein gene (*pfcsp*) which secreted by the sporozoite stage [5]. The *pfcsp* gene is known to be subdivided into 3 regions namely the N-terminal region, the central repeats region, and the C-terminal region. The N-terminal region is located at the 5′ end of PfCSP, comprised of around 84 to 104 amino acids (aa) [6]. The N-terminal region is also characterized by the presence of the KLKQP motif which is essential for the parasite entry to the hepatocytes. This along with the Th1R T-cell epitope which induces the antibody response in the human host [7]. For the C-terminal region which present at the 3′ end, it encompasses the polymorphic Th2R and Th3R T-cell sub-regions which are also been recognized by the human immune system [6]. Concerning the central repeats region, it is consisted of multiple motifs repeats of 4 aa. The NANP and the NVDP motifs were mostly seen among the different *P. falciparum* strains with NANP more frequent [8]. However, several other motifs were also reported from different geographical regions [9].

The currently used malaria vaccine, is comprised of the central repeat region and the T-cell epitope of the c-terminal region of the *P. falciparum* 3D7 together with hepatitis B virus surface antigen (HBsAg) [10]. The deployment of the RTS,S vaccine took place in a few African countries including Ghana, Malawi, and Kenya [11]. The vaccine showed variable immunization duration course that might last for up to 1 year; hence a booster dose is recommended especially in areas of high malaria multiplicity and intensity [12].

In Sudan, malaria is serious public health problem and under certain circumstances it develops to an epidemic level [13]. The national malaria control programme is challenged by the emergence and rapid development of both, drugs and insecticide resistance, urging the need for alternative tools for the disease control such as an effective vaccine [14, 15]. It occurs with variable severity onset and syndrome throughout the year, however, in some parts of the country malaria incidence is sporadic signifying the genetic diversity of the circulating *P. falciparum* and consequently promote the need to identify and characterize parasite populations that circulate in each specific region [14]. Therefore, this study is aimed at assessing the genetic diversity and population structure of the *P. falciparum* parasite in Khartoum state, Sudan, based on the *pfcsp* gene in order to assess congruency with different PfCSP reference strains together with different Sudanese isolates [16]. Moreover such data help in assess opportunity for the RTS,S vaccine successful deployment and evaluate effectiveness of the vaccine and aid in developing Sudan RTS,S vaccine [11].

## Methods

### Study sites and *Plasmodium falciparum* isolates

**Fig. 1:**
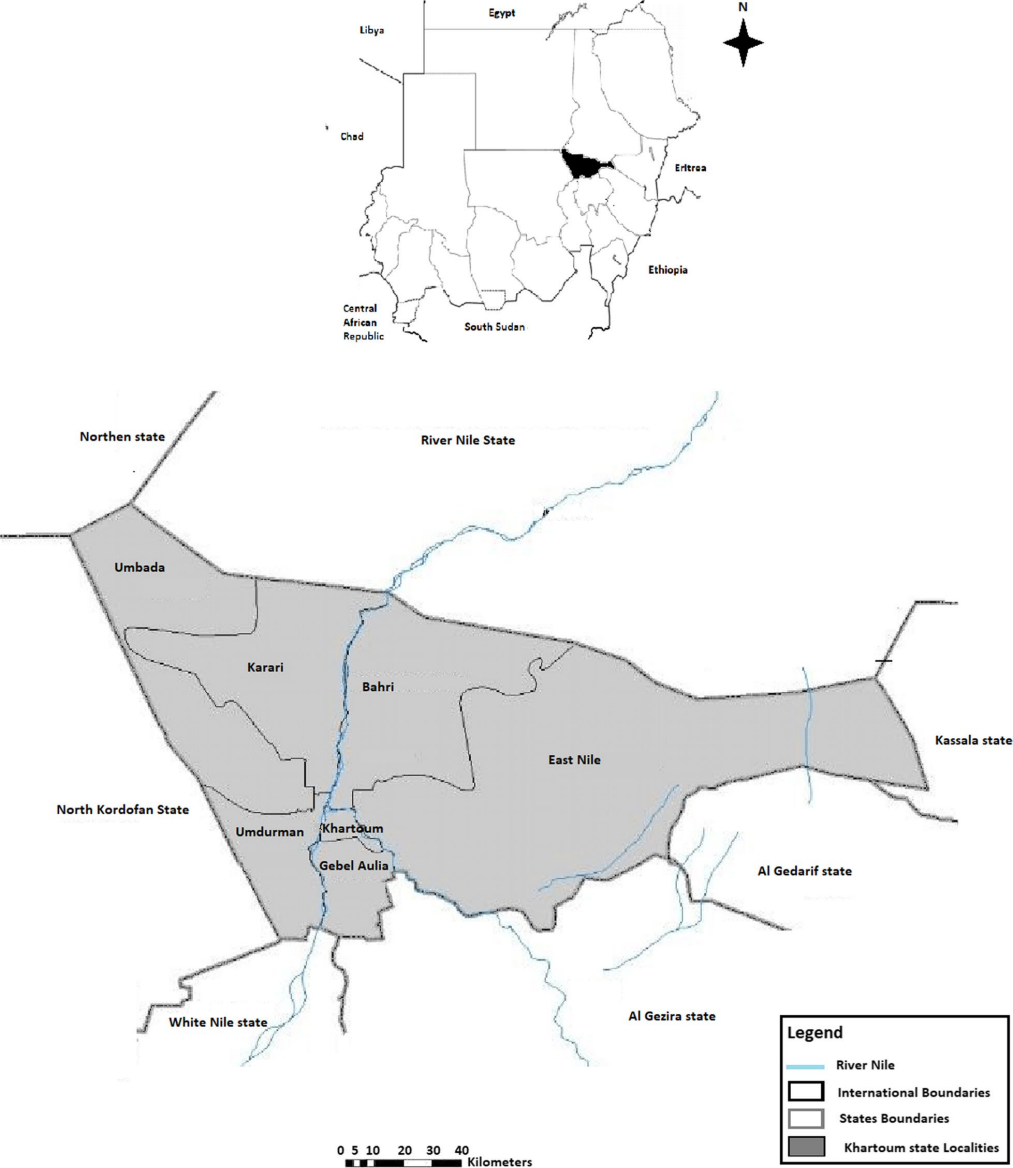
Map of Sudan. Showing the different Khartoum localities’ where *P. falciparum* isolates were collected

*Plasmodium falciparum* isolates were collected from different health centres in Khartoum State between January and December 2019. Khartoum state is one of the eighteen states of Sudan and considered as the capital of Sudan. It covers around 22.142 km^2^ and populated with around 7.688 million in 2017 censuses. Khartoum state is comprised of 7 localities; Khartoum, Omdurman, Bahri, Gebel Aulia, Karari, Umbada, and East Nile (Fig. 1). During the study period, a total of 261 febrile patients were recruited. Malaria diagnosis was made by expert microscopists using Giemsa-stained thick and thin blood films. Prior to samples collection, patients or legal guardians of children less than 18 years old were offered a detailed description of the study, and if they have voluntary accept to enroll in the study, they were requested to sign an inform consent to include their samples and information in the study. Demographic and clinical data were collected. The Clinical phenotyping of malaria infection were made according to WHO guidelines [17]. The collected blood samples were preserved at 4 °C until molecular examination. All methods followed for DNA extraction, Molecular detection and clonal infection determination of *Plasmodium* species, amplification and sequences analysis of the *pfcsp* gene were performed in accordance with the relevant guidelines and regulations.

### Molecular detection of *Plasmodium* species

The total genomic DNA of the parasite was extracted from patients’ blood samples using the QIAamp DNA Blood Mini Kit (Qiagen Inc. Germany). In order to confirm the microscopic results, all DNA samples were examined by the semi nested polymerase chain reaction (PCR) using the following primers; UNR: 5′ GAC GGT ATC TGA TCG TCT T 3′, PLF: 5′ AGT GTG TAT CAA TCG AGT TT 3′, and HUF: 5′ GAG CCG CCT GGA TAC CG 3′ for the first PCR reaction and PLF primer and falciparum-R: 5′ AGT TCC CCT AGA ATA GTT ACA 3′, vivax-R: 5′ AGG ACT TCC AAG CCG AAG 3′, ovale-R: 5′ GCA TAA GGA ATG CAA AGA ACA G 3′, and malariae-R: 5′ GCC CTC CAA TTG CCT TCT 3′, for the second PCR reaction to confirm the presence of *P. falciparum* mono-infection. PCR reaction was made using the Maxime™ i-Taq PCR premix kit (iNtRON Biotechnology, South Korea) according to the manufacturer instructions. Primers concentrations and thermocycling conditions were adjusted according to Rubio et al. [18].

### Molecular assessment of *P. falciparum* multiple clonal infections

In order to identify single clonal infection of the confirmed *P. falciparum* cases, the Merozoite Surface Protein 1 (*msp1*) gene was amplified to exclude multiclonal infections. Nested PCR was done using the following primers; outer msp1-f: 5′ CTA GAA GCT TTA GAA GAT GCA GTA TTG 3′ and outer msp1-r: 5′ CTT AAA TAA TAT TCT AAT TCA AGT GGA TCA 3′. Following the first outer reaction clonal primers were used including MAD20-f: 5′ AAA TGA AGA AGA AAT TAC TAC AAA AGG TGC 3′, MAD20-r: 5′ GCT TGC ATC AGC TGG AGG GCT TGC ACC AGA 3′, RO33-f: 5′ TAA AGG ATG GAG CAA ATA CTC AAG TTG TTG 3′, RO33-r: 5′ CAT CTG AAG GAT TTG CAG CAC CTG GAG ATC 3′, K1-f: 5′ AAA TGA AGG AAC AAG TGG AAC AGC TGT TAC 3′, and K1-r: 5′ ATC TGA AGG ATT TGT ACG TCT TGA ATT ACC 3′. The primers concentrations per each reaction and the PCR amplification conditions used were according to Ntoumi et al. [19].

### Amplification of the *pfcsp* gene

After the amplification of the *msp1* gene, *P. falciparum* were sorted out to exclude multiclonal infection since multiple clonal infections produce multiple products of *pfcsp,* which later give non-specific sequencing results. Genomic DNA of single clonal infections were further used for the amplification of *pfcsp*. The *pfcsp* gene amplification was done as previously published by Zeeshan et al. [9]; using the following primers; outer PfCSP-f: 5′ TTA GCT ATT TTA TCT GTT TCT TC 3′ and outer PfCSP-r: 5′ TAA GGA ACA AGA AGG ATA ATA CC 3′, for the first PCR reaction. The second PCR reaction was made using the primers; nested nPfCSP-f: 5′ GAA ATG AAT TAT TAT GGG AAA CAG 3′ and nPfCSP-r: 5′ GAA GGA TAA TAC CAT TAT TAA TCC 3′. PCR amplicons of the first run were used as a template for the second PCR. The amplified *pfcsp* products were visualized using agarose gel electrophoresis in comparison to a 100 base pair DNA ladder. Amplified PfCSP PCR products were further purified from gel using gel extraction kits and instructions were followed according to the manufacturer’s guidelines (New England Biolabs Inc, New England). Each amplicon was sent in replicate for sequencing by the Sanger dideoxy sequencing method using the primer SeqPfCSP: 5′ TGG GTC ATT TGG CAT ATT GTG 3′ using ABI3500 sequencer (Applied Biosystems SeqStudio, 3500 series) provided by Macrogen Inc. (The Netherlands).

### pfcsp sequence analysis

The obtained sequences of the PfCSP amplicons were checked for the correctness of sequences reads and nucleotides base calling errors by aligning of the forward and the complementary reverse sequence using GENtle software (v1.9.4) [20]. Sequences were also trimmed at the primers sequences reads to reduce sequencing mismatching that occurs at the beginning of the sequencing process. Using the NCBI GenBank database, all worldwide previously published PfCSP sequences were included in the analysis of the comparison with the Sudanese sequences similarity and divergence (https://www.ncbi.nlm.nih.gov/nuccore). Local Sudanese sequences comparison and divergence were made on the N-terminal, C-terminal and the NANP repeat regions of the PfCSP gene. Reference sequences of the *pfcsp* gene including the 3D7 (XM_001351086) [21], NF54 (M22982.1) [22], 7G8 (AB121015.1), HB3 (AB121018.1), Dd2 (AB121017.1), MAD20 (AB121020.1), and RO33 (AB121021.1) [23], and the Wellcome strain (M15505.1) [24]. Using MEGA7 software, the nucleotide substitution model with the least Bayesian Information Criterion (BIC) score was considered as a best fit model to conduct the phylogenetic tree [25]. The phylogenetic tree was conduct to investigate the relationship of the Sudanese PfCSP sequences with the PfCSP reference sequences. Hasegawa-Kishino-Yano with Gamma distribution model (HKY + G + I) was used for creating the tree [26]. Using the software DnaSP (v5.10) [27], nucleotides diversity parameters including the numbers of haplotypes (Hap), haplotypes diversity (Hapd), the average number of nucleotide differences between two sequences (P), and the numbers of segregating sites (S) were obtained. To investigate the genetic relationship of the Sudanese PfCSP sequences with the worldwide PfCSP sequences, and since the central repeat region is an immunodominant epitope of PfCSP, and it had been applied to the component of RTS,S malaria vaccine, the haplotypes network was constructed using the online tcsBU software [28] Natural selection theory was also tested on the C-terminal region of PfCSP using Fuand Li’s D, Fuand Li’s F statistics, and Tajima’s D test using DnaSP [29, 30] to investigate the role of immunity in the initiation of sequence divergence at the Th2 and Th3 located at the C-terminal region.

### Statistical analysis

Data analysis was made using the Statistical Package for Social Sciences (SPSS version 20). Chi-Square test was calculated to test the significance association between the msp1 gene and the presence of the insertion at the N-terminal region, and the number of NANP repeats at the Central repeat region. A *p* value < 0.05 was considered a statistically significant.

## Results

### Genotyping and assessment of *P. falciparum* multiple clonal infections

The study included a total of 261 febrile patients consisted of 143 (54.8%) male and 118 (45.2%) females. All study participants have reported fever > 37.5 °C. Based on the WHO classification, all patients were classified as mild uncomplicated malaria infection. The microscopic examinations revealed that a total 196 (75.1%) patients were positive for *P. falciparum* infection whiles the remaining 65 (24.9%) were harbouring co-infection of *P. falciparum*/*Plasmodium vivax*. Molecular analysis using the semi-nested PCR confirmed the presence of 23.9% (47/196) *P. falciparum*/*P. vivax* co-infections. Confirmed cases of mono-infections of *P. falciparum* were further amplified for the *msp1* gene to exclude multiple allelic infections which will later affect the sequencing process of the *pfcsp* gene. Out of the 149 *P. falciparum* mono-infections, *msp1* revealed the presence of 83 (55.7%) multiple allelic infection which were excluded from the amplification process of the *pfcsp* gene. The selected 66 (44.3%) *P. falciparum* single allelic, mono-infections isolates were amplified for the *pfcsp* gene. PCR amplification produced product sizes that vary in length from 1 kb to 1.2 kb.

### Phylogenetic network of the Sudanese *pfcsp* and *pfcsp* reference sequences

**Fig. 2:**
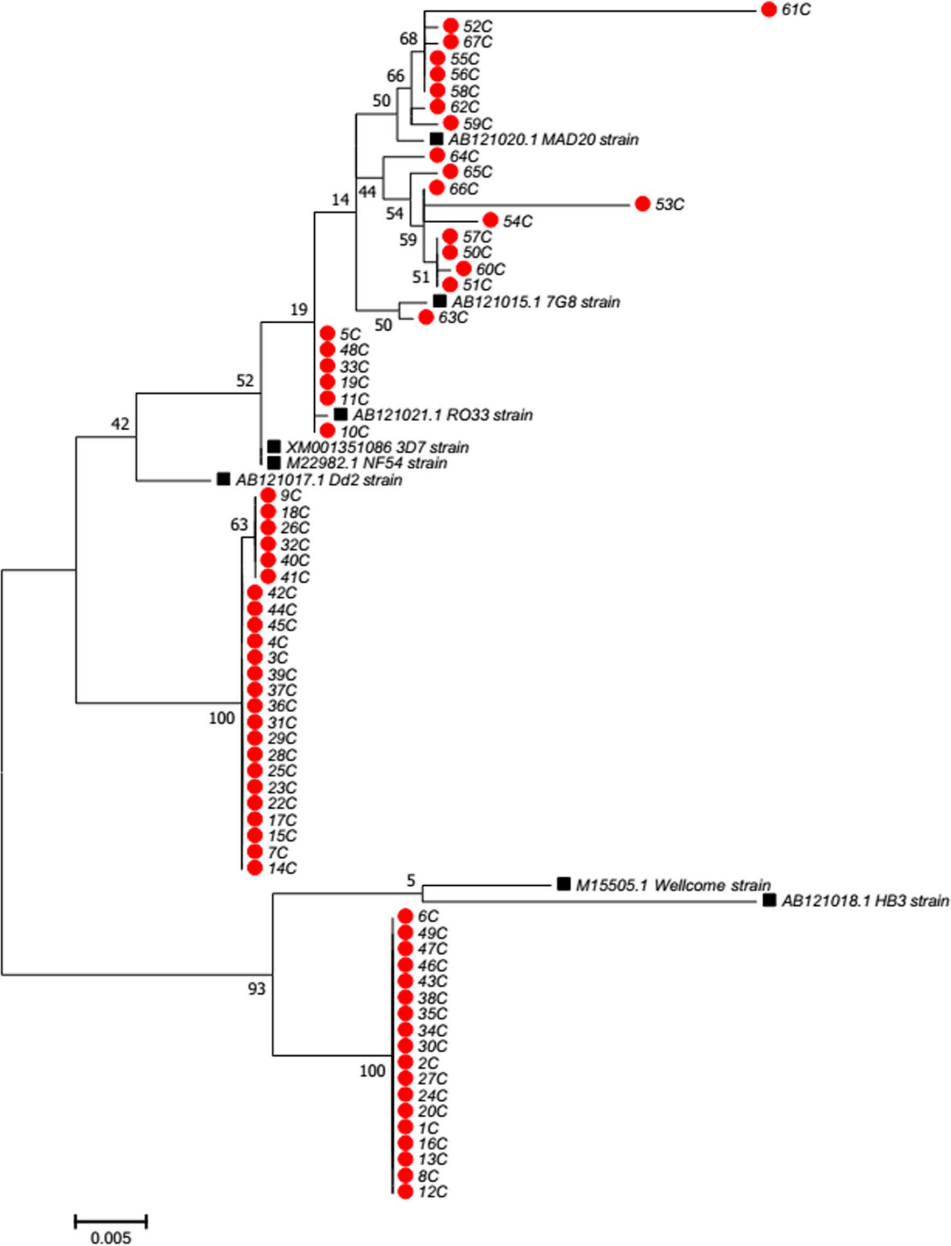
Phylogenetic network of the complete Sudanese PfCSP sequences and the PfCSP reference sequences. Red circles correspond the Sudanese PfCSP sequences. Black boxes correspond the PfCSP reference sequences. 3D7 (XM_001351086), NF54 (M22982.1), 7G8 (AB121015.1), HB3 (AB121018.1), Dd2 (AB121017.1), MAD20 (AB121020.1), and RO33 (AB121021.1), and the Wellcome strain (M15505.1)

In comparison with the different PfCSP reference strains, the Sudanese isolates showed a considerable similarity with other African isolates. The phylogenetic analysis showed that most of the sequences clustered together forming a major 2 different clades consisting of the majority of the study isolates, also withing the two major clades, sequences divergence was noticed. The first clade consisted of 24 isolates of them 18 isolates were clustered together with a bootstrap value of 100% similarity and the other 6 isolates clustered together with a bootstrap value of 63. For the second major clade, 18 isolates clustered together with a bootstrap value of 100% and showing slight similarity with the Wellcome and the HB3 reference strains. The remaining isolates were distributed forming 2 different smaller clades with a considerable similarity with the 3D7, MAD20, RO33, Dd2, 7G8, and the NF54 reference strains (Fig. 2).

### Genetic diversity in the N-terminal region of the Sudanese and global *pfcsp*

**Fig. 3:**
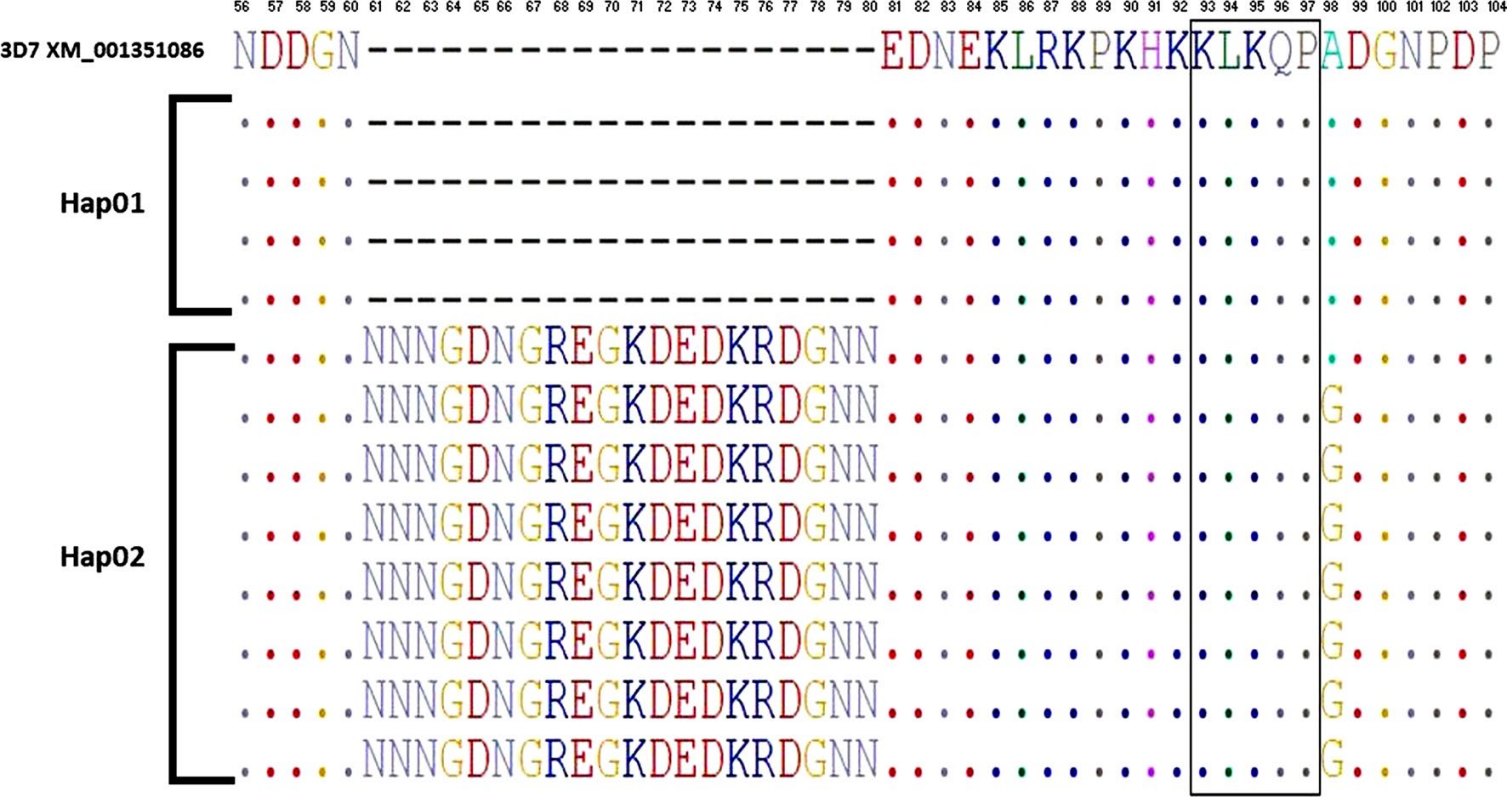
Sequence diversity in the PfCSP N-terminal region of the Sudanese isolates. Comparison was made in relation to the *P. falciparum* reference sequence 3D7 (Accession No: XM_001351086). The dashes present the insertion NNNGDNGREGKDEDKRDGNN, which is present only in samples belonging to Hap02. Hap01 was consisted of 19 isolates, and Hap02 consisted of 47 isolates. Sequences shown are representative sequences to the total sequences investigated to present the insertion. KLKQP motif indicated with a rectangular is conserved through all the study isolates

The analysis of the N-terminal region showed the presence of 2 different haplotypes; Hap01 included the 3D7 reference strain plus 19 isolates, and Hap02 included 47 isolates. The haplotype diversity score was 0.425 ± 0.00727. The low haplotype diversity among the Sudanese isolates indicates that the N-terminal region is highly conserved. The 2 haplotypes were observed due to the amino acid polymorphism at A98G. The amino acid insertion “61NNNGDNGREGKDEDKRDGNN80” was detected in a total of 47 isolates representing Hap02. Whereas the KLKQP motif was conserved in all the isolates (Fig. 3).

### Genetic diversity in the central repeat region of the Sudanese *pfcsp*

**Fig. 4:**
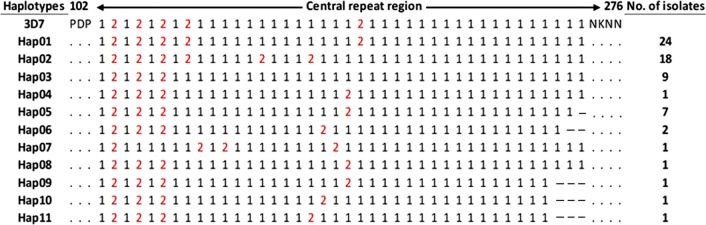
Sequence diversity in the central repeat region of the Sudanese PfCSP gene. Comparison was made in relation to the *P. falciparum* reference sequence 3D7 (Accession No: XM_001351086). 1 represents NANP motif, 2 represents NVDP motif. Of the 66 sequences investigated, numbers of isolates per each haplotype detected are indicated

Based on the NANP and NVDP repeats of the central repeat region, a total of 11 haplotypes were seen with a Hapd of 0.779 ± 0.00097. Hap01 and Hap02 were the most frequent haplotypes; 24 isolates and 18 isolates, respectively, followed by haplotypes Hap03, Hap05, and Hap06 which consisted of 9, 7, and 2 isolates, correspondingly. Whereas the remaining haplotypes consisted of a single isolate per each. The numbers of NANP repeats at each haplotype were ranging from 33 to 37 repeats whereas NVDP repeats were ranging from 3 to 6 repeats. Accordingly, the total number of repeats per haplotype was ranging from 37 to 40 repeats (Fig. 4).

### Association of *msp-1* genotyping with sequences diversities at the N-terminal and the central repeat regions of the Sudanese *pfcsp* genes

Analysis of sequences diversity at the N-terminal and the central repeat regions of the PfCSP gene in relation with the different *msp1* genotypes did not show any significant association between the different msp1 genotypes and the presence of insertion in the N-terminal region nor the number of NANP motif repeats of the PfCSP gene, *p* values > 0.05 (Table 1).

**Table 1:** Association of MSP-1 genotyping with sequences diversities at the N-terminal and the central repeat regions of the Sudanese PfCSP genes

MSP-1 genotype	Sample origin	Presence of insertion in the N-terminal region of PfCSP		Total	P value	Number of NANP motif repeats					Total	P value
		Yes	No			33	34	35	36	37		
MAD20	Khartoum	6	–	6	0.428	–	–	6	–	–	6	0.787
	Bahri	1	–	1		–	–	1	–	–	1	
	East Nile	1	–	1		–	–	1	–	–	1	
	Gebel Aulia	1	1	2		–	–	2	–	–	2	
	Karari	1	–	1		–	–	1	–	–	1	
	Omdurman	1	–	1		–	–	1	–	–	1	
	Umbada	1	–	1		–	–	1	–	–	1	
K1	Khartoum	5	2	7	0.361	–	5	2	–	–	7	0.440
	Bahri	2	–	2		–	–	2	–	–	2	
	Gebel Aulia	3	2	5		–	3	2	–	–	5	
	Karari	2	–	2		–	–	2	–	–	2	
	Omdurman	2	3	5		–	2	2	–	1	5	
	Umbada	3	–	3		–	1	2	–	–	3	
RO33	Khartoum	4	6	10	0.358	–	4	2	2	2	10	0.763
	Bahri	–	–	0		–	–	–	–	–	0	
	East Nile	5	1	6		2	1	1	–	2	6	
	Gebel Aulia	2	2	4		–	1	1	–	2	4	
	Karari	4	1	5		–	3	–	1	1	5	
	Omdurman	1	–	1		–	–	1	–	–	1	
	Umbada	1	2	3		1	–	1	–	1	3	
Total		46	20	66		3	20	31	3	9	66	

### Diversity patterns in the C-terminal region of the Sudanese *pfcsp*

**Fig. 5:**
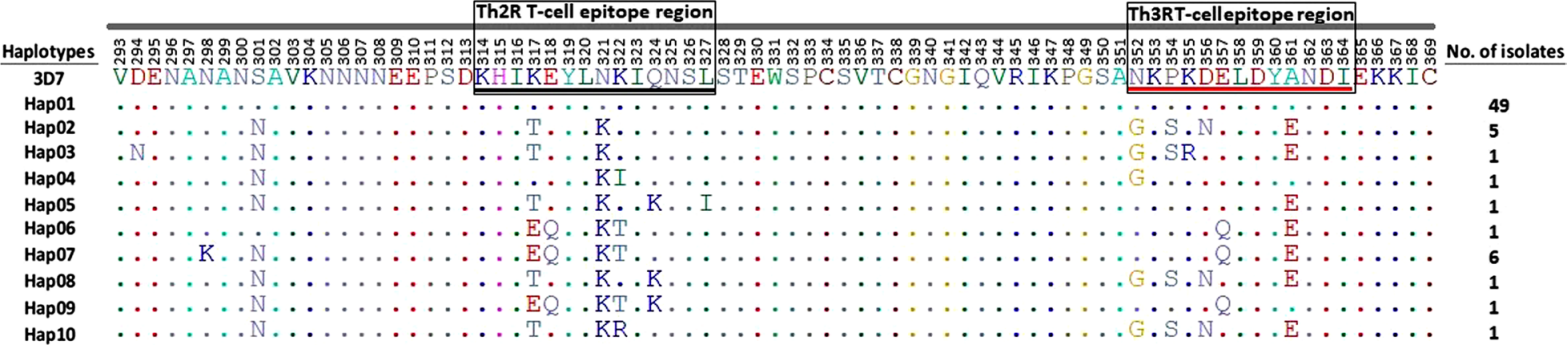
Sequences diversity patterns in the C‑terminal region of the Sudanese PfCSP gene. Comparison was made in relation to the *P. falciparum* reference sequence 3D7 (Accession No: XM_001351086). Dots represents similar amino acids in comparison to the reference sequence. Of the 66 sequences investigated, numbers of isolates per each haplotype detected are indicated

The analysis of the genetic diversity in the C-terminal region of the Sudanese *pfcsp* gene showed the presence of 10 different haplotypes with a haplotype diversity of 0.457 ± 0.073. Hap01 was the most frequent haplotype; 49 isolates. Hap02 was reported among 5 isolates, Hap07 was reported among 6 isolates. the remaining haplotypes were only represented by one isolate each (Fig. 5). Non-synonymous substitutions were seen in different amino acids in the C-terminal region. D294N, K298N, and N301S, were seen in Hap03, Hap07, and all the haplotypes except for Hap01 and Hap06, respectively. Several non-synonymous amino acids changes were also seen at the Th2R and the Th3R T-cell epitope regions including T317K, E317K, Q318E, K321N, I322K, T322K, R322K, K324Q, I327L, G352N, S354P, R355K, N356D, Q357E, and E361A (Fig. 5). The analysis of the C-terminal nucleotide diversity was 0.00929 ± 0.00150, and an average number of nucleotide differences of 3.51199 was observed. Neutrality testing indices to investigate the null hypothesis of natural selection using Fu and Li’s D*, Fu and Li’s F*, and Tajima's D tests were − 0.40167, − 0.45855, and − 0.35629. All tests were statistically not significant, P > 0.10.

### Haplotype network analysis and diversity patterns in the C-terminal region of the Sudanese and global PfCSP

**Fig. 6:**
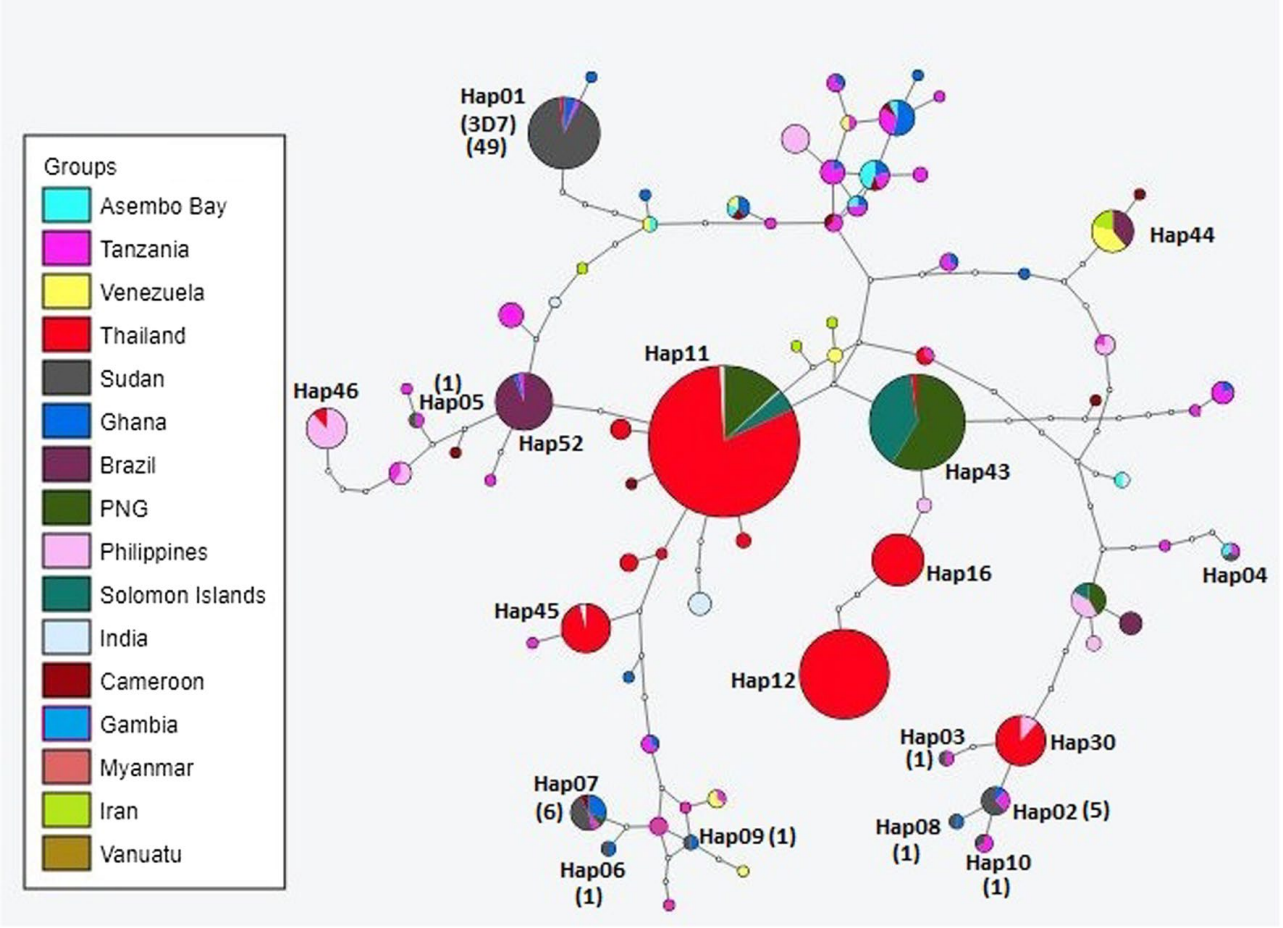
Haplotype network analysis and sequences diversity patterns in the C‑terminal region of the Sudanese and global *pfcsp* gene. Haplotypes numbers of the main haplotypes are indicated at each node. The number of sequences of the Sudanese isolates are indicated in brackets next to their representative haplotype

Based on the C-terminal region, the analysis of the haplotype network of the Sudanese isolates and the global published C-terminal region of the PfCSP the number of haplotypes detected was 69 different haplotypes with a haplotype diversity of 0.8651 ± 0.008. Hap11 was the most frequent haplotype, followed by Hap43, Hap12. The haplotype that includes the 3D7 reference strain was considered Hap01. Hap01 was including most of the studied Sudanese isolates; 48 isolates out of 66 isolates. The remaining isolates were distributed into Hap02 (5 isolates), Hap03 (1), Hap04 (1), Hap05 (1), Hap06 (1), Hap07 (6), Hap08 (1) Hap09 (1), and Hap10 (1) (Fig. 6).

## Discussion

In Khartoum State, *P. falciparum* is accounted for up to 90% of malaria infections, while 10% are acknowledged to be caused by *P. vivax*. Investigating the genetic diversity of the Sudanese *P. falciparum* is extremely important to further warrant the deployment of the RTS,S malaria vaccine [16]. The aim of this study was to investigate the genetic diversity of *pfcsp* gene in Sudan. In this study, the investigated *pfcsp* gene showed a high conversation at the N-terminal region when compared to the 3D7 reference strain. Although, a higher conservation is reported in this study in which only 2 haplotypes were detected compared to previous studies which could be attributed to the difference in location which was reported from different regions of Sudan and also the sample size included, where they only investigated 21 *P. falciparum* N-terminal region sequences, and specifically only 4 sequences from Khartoum were investigated [16]. Also, the presence of the other unique polymorphisms can be certain to each specific location depending on the circulating *P. falciparum* strain. The (NNGDNGREGKDEDKRDGNN) insertion which was not reported previously could be mainly due to the small sample size been investigated which led to the misdetection of this insertion previously which is noticeable by the small frequency of the insertion reported in this study. Meanwhile, this insertion has been detected in several other geographical regions, such as Myanmar [8].

Although, the functional role of this insertion when it’s present or absent is limited investigated and not fully understood yet. Although analysis of the different *msp1* genotypes with sequences diversities at the N-terminal region of the Sudanese *pfcsp* gene did not reveal any association for the presence of the insertion. therefore the presence of the insertion could be explained in terms of different parasite strains, geographical or environmental adaptation of the parasite, or it might be present as a form of parasite adaptation to the host immune response which is mainly targeted at the Th1R T-cell epitope present at the N-terminal region [7]. Accordingly, the Th1R T-cell epitope at the N-terminal region is showing relatively high polymorphisms, this can be explained as a role of parasite adaptation to host immune system. This was previously investigated by Conway et al. when a monoclonal antibody anchored to the linear T-cell epitope “81EDNEKLRKPKH91” which is located before the KLKQP motif which is responsible for sporozoite entry to the hepatocytes [31]. The monoclonal antibody effectively neutralized the infectivity of the sporozoite in-vivo [31]. Also, Bongfen et al. demonstrated that the development of polypeptides that flank the N-terminal region played a significant role in the initiation of inhibitory antibodies production to prevent sporozoite invasion to the hepatocytes [32]. Thus, supporting the hypothesis that the N-terminal region can play an important role as a vaccine candidate which is aided by the high conservation rate [31].

This study has detected a high haplotype diversity at the central repeat region, this in turn has highlighted the genetic diversity of this region is mainly sustained by balancing natural selection due to the effect of human immune response [31]. This also indicated by the negative, statistically insignificant values of Fu and Li’s D*, Fu and Li’s F*, and Tajima’s D tests. However, bottleneck effect and population expansion are both scenarios cannot be neglected completely. Interestingly, as the difference in the NANP and NVDP repeats controls the difference in the *pfcsp* gene length, the numbers of NANP repeats detected among this studied isolates were lower than previously reported by Lê et al. [8] and Zeeshan et al. [9], where higher numbers of NANP repeats was detected as well as several unique repeat motifs including NANS, NTNP, NVVP, NAHP, NAKP, NVNP, NAIP, NANL, NPNP, NVAD, NADP, SANP, and KANP. However, this variation in the central repeat region requires more research to understand the effect of these variation in terms of parasite genetic signature and host immune response. Previously, the central repeat region has been found to affect the stability of the *pfcsp* gene, which is suggested to be a copy-number dependent and further growths by the increase in the number of repeats [33]. The Sudanese isolates showed a relatively low numbers of repeats that ranged from 37 to 40 repeats similar to the African and South American PfCSP; 36 to 37 repeats, while considerably lower than the Asian PfCSP; 40 to 43 repeats [8]. This indicates that the difference in the numbers of the tetrapeptide repeats at the central repeat region could be attributed to geographical distribution, host (human and/or mosquito vectors) population genetic differences, or the different selection pressure imposed by the different case management protocols/drugs in use that resulted in form of co-evolution response to maintain parasite stability at specific origin. Also, the central repeat region is important in the RTS,S malaria vaccine. The RTS,S consisted of 19 tetrapeptide repeats of the NANP repeat [34]. However, still no reports indicated that the different repeats number can or may affect the effectiveness of the RTS,S vaccine (see Fig. 6).

Investigating the genetic diversity of the C-terminal region of the Sudanese *pfcsp* gene showed a relatively low genetic diversity expressed by the presence of only 10 haplotypes, with Hap01 to be the most frequent; 49 isolates, and it has two insertions; the “KHIEKEYLNKIQNSL” in the Th2R and “NKPKDELDYANDI” in the Th3R regions T-cell epitopes. This haplotype has been also prevalent among other African PfCSP isolates, this is further explained by the haplotype network where most of the Sudanese isolates clustered with the Ghanaian isolates. The low haplotype diversity detected among the Sudanese PfCSP isolates indicates a low level of genetic diversity. Since the RTS,S malaria vaccine is composed of the C-terminal T-cell epitope, this region can be very important in terms of designing regional specific vaccines based on identifying the circulating *P. falciparum* strains in that specific region [34].

The analysis of the global C-terminal region haplotype network indicated a unique clustering of the Asian isolates in a relatively separated haplotypes from the African and the South American isolates. This has been also discussed previously by Lê et al., where African isolates presented with branched and complex patterns of haplotypes [8]. This also supports the suggestion that African PfCSP is more diverse than the PfCSP of the other geographical regions.

Since the deployment of the RTS,S malaria vaccine is mainly controlled by understanding and identifying the genetic diversity of the circulating *P. falciparum* strains in a certain geographical region [11]. RTS,S vaccine trials showed a significant effect in reducing malaria incidence in many African countries including Gambia, Mozambique, Ghana, Tanzania, Gabon, and Kenya [35–37]. Therefore, this study findings are showing the higher prevalence of a haplotype, which is more similar to the 3D7 reference strain and also worth mentioning that the current RTS,S is primarily constructed from the PfCSP of the 3D7 strain, the deployment of the RTS,S malaria vaccine clinical trials can be effective in reducing malaria incidence in Sudan.

Although, this study investigated the genetic diversity of *P. falciparum pfcsp* gene in Sudan, which is currently part of the RTS,S malaria vaccine formulation. However, despite the importance of mapping genetic diversity, the results are still at the level of simple gene sequence observations for the identification of sequence changes which might be validated for what concerns their impact on vaccine efficacy. The effects on parasite fitness are neither tested at the cell nor biochemical levels. Therefore, further experiments to establish how these changes impact parasite and/or host biology.

## Conclusion

This study is the first study to investigate the genetic diversity of *P. falciparum* in Sudan based on studying the complete *pfcsp* gene. This study was limited to investigating the genetic diversity in specific region in Sudan; Khartoum. Therefore, further studies are warranted to explore the genetic diversity of PfCSP in the whole country since Sudan is known with its large geographical space that has different environmental conditions and endemicity situations that might influence the development of different adaptive evolutionary traits among the parasite population increasing its genetic diversity. Also, there are potential evolutionary consequences could not be observed in this study samples were co-infections nor multiclonal infections were investigated. The combination of these factors might impact the effectiveness of the RTS,S vaccine if been deployed in Sudan or areas with similar scenario, that why a more comprehensive study with a larger sample size that collected from different regions is highly recommended. The *pfcsp* gene is considered a core element of the RST,S malaria vaccine that currently been used, nevertheless, the genetic diversity in the *pfcsp* gene among the different regions *P. falciparum* strains may affect the efficacy of the RTS,S vaccine. In this study, the analysis of the genetic diversity of the PfCSP population structure indicated that the *pfcsp* gene in Sudan is highly conserved with few genetic variations reported at the central repeat region and the C-terminal region. Meanwhile, the limited diversity reported at the N-terminal region postulates the usefulness of this region in the development of new vaccine candidate.

## Data Availability

All datasets generated for this study are included in the manuscript. Sequences generated in this study are available at the NCBI GenBank database under the accession numbers; MW554526-MW554591.

## Abbreviations

Hap: Haplotype
Hapd: Haplotype diversity
Msp1: Merozoite Surface Protein 1
P: Average number of nucleotide differences
PfCSP: Circumsporozoite surface protein of *Plasmodium falciparum*
S: Segregating sites
